# Effect of Linoleic Acid on Cholesterol Levels in a High-Fat Diet-Induced Hypercholesterolemia Rat Model

**DOI:** 10.3390/metabo13010053

**Published:** 2022-12-30

**Authors:** Nurul Adila Azemi, Ahmad Khusairi Azemi, Luqman Abu-Bakar, Vigneswari Sevakumaran, Tengku Sifzizul Tengku Muhammad, Noraznawati Ismail

**Affiliations:** 1 Institute of Marine Biotechnology, Universiti Malaysia Terengganu, Kuala Terengganu 21030, Terengganu, Malaysia; 2 Faculty of Veterinary Medicine, Universiti Malaysia Kelantan, Kota Bharu 16100, Kelantan, Malaysia

**Keywords:** cardiovascular disease, linoleic acid, fenofibrate, obesity, hypercholesterolemia, HDL, LDL

## Abstract

Cardiovascular disease is the leading cause of morbidity and mortality worldwide, accounting for almost one-third of all deaths. The risk factors for developing this disease include high levels of serum total cholesterol (TC), triglycerides (TG), and low-density lipoprotein (LDL), alongside low levels of high-density lipoprotein (HDL). Dietary linoleic acid has been suggested to reduce these risk factors. This study aims to determine the effects of linoleic acid on cholesterol levels, liver function tests, and structural changes in liver tissue in comparison with fenofibrate in a hypercholesterolemic rat model. Thirty-six male Sprague Dawley rats (150–180 g) were divided into non-hypercholesterolemic and hypercholesterolemic groups. Hypercholesterolemia was induced in the rats by feeding them with a high-fat diet for two weeks. After two weeks, the non-hypercholesterolemic and hypercholesterolemic rats were equally divided into six groups (*n* = 6): control non-hypercholesterolemic rats, non-hypercholesterolemic rats treated with fenofibrate (60 mg/kg), non-hypercholesterolemic rats treated with linoleic acid (5 mg/kg), control hypercholesterolemic rats, hypercholesterolemic rats treated with fenofibrate (60 mg/kg), and hypercholesterolemic rats treated with linoleic acid (5 mg/kg). The changes in the rats’ body weight, serum lipid profiles, atherogenic indices, and liver function test results were obtained. The rats’ liver tissues were stained for histopathological analysis. The linoleic acid-treated hypercholesterolemic rats exhibited significantly reduced serum TC, TG, LDL, aspartate aminotransferase, and alanine aminotransferase levels, as well as increased HDL levels compared with the control hypercholesterolemic rats. These linoleic acid effects were comparable to those in the fenofibrate-treated hypercholesterolemic rats. In conclusion, linoleic acid possesses early anti-hypercholesterolemic properties, which may be due to the reductions in serum cholesterol levels and mild early structural changes in the liver tissues of hypercholesterolemic rats. Therefore, continued studies on linoleic acid in atherosclerotic and/or obese animal models are suggested.

## 1. Introduction

Cardiovascular disease is the leading cause of death worldwide. It gives rise to about 18.6 million annual deaths, which accounts for almost one-third of all deaths [1]. The underlying pathogenesis and progression associated with nearly all cardiovascular diseases are predominantly of atherosclerotic origin, leading to the development of coronary heart disease, peripheral vascular disease, venous thromboembolism, and cerebrovascular disease, subsequently causing myocardial infarction and stroke. The etiological risk factors leading to the onset of cardiovascular diseases are well recognized and include hyperlipidemia, obesity, diabetes, hypertension, smoking, and lack of physical activity. Atherosclerosis, which is caused by cholesterol metabolic disorders and chronic inflammation, is the major pathology for cardiovascular diseases [2,3].

Hypercholesterolemia, particularly the augmented low-density lipoprotein (LDL) cholesterol level, is closely related to the risk of cardiovascular diseases, as LDL cholesterol indicates an increased risk. Meanwhile, high-density lipoprotein (HDL) cholesterol is considered a protective factor. Both LDL and HDL are independent risk factors for cardiovascular disease [4]. HDL is a part of the diagnostic criteria for metabolic syndrome and an important target for the treatment and prevention of cardiovascular diseases [5,6,7]. The main modality of the anti-atherosclerosis function by HDL is through reverse cholesterol transport, which is an important part of cholesterol and lipoprotein metabolism. This process involves the transport of excessive cholesterol from the peripheral tissue to the liver for recirculation or excretion, which can reduce lipid deposition on the blood vessel wall [5,6,7,8].

Linoleic acid (18:2n-6) is an essential polyunsaturated fatty acid (PUFA) that is widely found in plant oil. In humans, linoleic acid cannot be synthesized and must be acquired through diet [9,10]. Linoleic acid is used to synthesize a variety of other unsaturated fatty acids, including eicosapentaenoic acid and docosahexaenoic acid. Previously, these unsaturated fatty acids have been shown to improve blood pressure, platelet reactivity, thrombosis, triglyceride (TG) levels, vascular reactivity, heart-rate variability, and inflammation [11,12,13]. In another study, linoleic acid was reported to reduce blood cholesterol and play a significant role in preventing cardiovascular diseases [14]. This unsaturated fatty acid may exert its LDL-C lowering properties by increasing membrane fluidity, which increases LDL receptor activity and consequently, decreases LDL apoB and increases LDL catabolism [15,16,17]. In addition, it increases CYP7 activity, thereby converting cholesterol to bile acids in the liver. This mechanism will likely indirectly increase LDL receptor production [15]. In other studies, linoleic acid consumption has been reported to increase HDL levels [18,19]. The increase in HDL levels is due to an increase in apolipoprotein A1 (ApoA1) expression [17,20]. ApoA1 triggers a reaction called cholesterol esterification that converts cholesterol to a form that can be fully integrated into HDL and subsequently transported through the bloodstream from the body’s tissue to the liver [21,22,23].

Fenofibrate is a potent lipid-lowering drug for treating hyperglyceridemia and mixed hyperlipidemia. It efficiently reduces plasma TG and LDL cholesterol and increases HDL cholesterol [24] by upregulating the hepatic gene expression and synthesis of ApoA1, the major apolipoprotein of HDL cholesterol. Previously, some studies have reported that fenofibrate shows superiority in raising HDL cholesterol compared with simvastatin and atorvastatin [24,25]. Fenofibrate exerts its activity via the activation of the nuclear receptor peroxisome proliferation-activated receptor α (PPARα). PPARα, in conjunction with the retinoid X receptor, positively regulates the transcription of the target gene by binding to a specific gene promoter response element. This mode of action is particularly important for the regulation of genes that control lipid and lipoprotein metabolism and may largely explain the normolipidemic action of fenofibrate [26].

Studies have observed that disorders induced by high-fat diets resemble human metabolic syndrome, with implications for cardiovascular health [27]. A high-fat diet is a high-density energy diet that provides 30% to 60% of calories from fat and is widely used in experimental animal models to induce obesity associated with insulin resistance, hypercholesterolemia, and atherosclerosis [28]. Previously, a high-fat diet has been used to induce or accelerate the formation of atherosclerotic lesions in animal models [29,30]. In addition, this diet negatively affects the sensitivity of the insulin receptors and the expression of the intracellular glucose transporter GLUT4, thereby contributing to the insulin resistance of the peripheral tissue [28,31,32].

Currently, there is a dearth of studies on the effect of linoleic acid on hypercholesterolemia in animal models. Hypercholesterolemia is the predominant metabolic disorder type in humans. Thus, this study aims to evaluate the effects of dietary linoleic acid on cholesterol levels, liver function tests, and early structural changes of the liver tissue in comparison with the lipid-lowering agent fenofibrate in a high-fat-diet-induced hypercholesterolemia rat model.

## 2. Materials and Methods

### 2.1. High-Fat Diet Preparation

A high-fat diet was prepared according to the method previously described by Lassoued et al. [33], with some modifications. The high-fat diet was prepared from a mixture of a 78.9% standard mouse diet (normal diet), 1% cholesterol powder, 0.1% cholic acid, 15% corn starch, and 5% corn oil.

### 2.2. Experimental Animals

Thirty-six male Sprague Dawley rats weighing between 150 and 180 g were used for the study. The rats were maintained under control conditions (22 ± 2 °C, 12 h light/12 h dark cycle) and fed a normal diet and water ad libitum. After acclimatizing, the rats were divided into hypercholesterolemic and non-hypercholesterolemic groups. The rats in the hypercholesterolemic group were fed a high-fat diet, while the non-hypercholesterolemic rats were fed a normal diet. The rats in both groups were fed with their respective diets for two weeks. The high-fat diet was administered for two weeks to develop hypercholesterolemia in the rats, in accordance with previous studies [34,35]. In the third week, both hypercholesterolemic and non-hypercholesterolemic rats were divided into three sub-groups (*n* = 6), as shown in Table 1.

The treatments were given via oral gavage for 28 days [36,37,38]. The doses of linoleic acid and fenofibrate used in the present study were according to previous studies [39,40]. Blood samples were collected from the tail vein and used for serum lipid profile and liver function analysis. The rats were then sacrificed through intraperitoneal (i.p.) injections of 300 mg/kg ketamine and 30 mg/kg xylazine [41]. The liver was dissected for histopathological analyses. The liver weight was measured at the end of the study period. The current study was conducted according to the guide for the Care and Use of Laboratory Animals, Universiti Malaysia Terengganu Animal Ethics Committee (Reference No. UMT/JKEPHT/2018/14).

### 2.3. Biochemical Measurements of Serum Lipid Profile and Liver Function Test

#### 2.3.1. Serum Lipid Profile and Atherogenic Index (AI)

A total of 700 μL of the blood sample was centrifuged at 6000 rpm (Eppendorf Centrifuge 5810R) for 20 min, and the resulting serum was used for lipid profile analyses. Serum total cholesterol (TC), TG, and HDL were measured, as described by Azemi et al. [27] and Ozturk et al. [42]. Serum LDL was calculated using the Friedewald formula [27,42] as follows: LDL = TC − (HDL + TG/5). The AI was calculated as LDL/HDL [27].

#### 2.3.2. Liver Function Test

Aspartate aminotransferase (AST) levels were measured using a Rat AST ELISA assay kit (Catalog No. E-EL-R0076) purchased from Elabscience (Houston, TX, USA), and the testing was performed according to the procedures detailed in the kit. Alanine aminotransferase (ALT) levels were measured using a Rat ALT ELISA assay kit (Catalog No. E-BC-F038) purchased from Elabscience (Houston, TX, USA), and the testing was performed according to the procedures detailed in the kit.

### 2.4. Histopathology

The histopathological study was carried out according to the method described by Zakaria et al. [43]. Liver tissue samples were fixed in 10% neutral formaldehyde. The specimens were dehydrated through an ascending series of alcohols, cleared in xylene using an automated tissue processor (Leica TP 1020, Nussloch, Germany), and then embedded in paraffin wax. Sections were cut at 5 μm using a rotary microtome, and then mounted on the glass slide. The sections were deparaffinized with xylene and rehydrated by decreasing the ethanol concentrations. The sections were then stained with hematoxylin and eosin and subsequently examined using a light microscope with a digital camera attachment at ×400. The histology sections were subjected to semi-quantitative analysis, in which the steatosis of the liver was scored using the NASH Clinical Research Network Scoring System [43,44]. The steatosis was graded on a scale of one to four with respect to the presence of cholesterol droplets. The hepatic steatosis was graded based on the percentage of fat within the hepatocytes as follows: Grade 0 (healthy; <5%), Grade 1 (mild; 5–33%), Grade 2 (moderate; 34–66%), and Grade 3 (severe; 66%>) [45].

### 2.5. Statistical Analysis

Statistical analysis was carried out using GraphPad Prism software (San Diego, CA, USA). Group comparisons were assessed via one-way analysis of variance with post hoc multiple comparisons using Tukey’s test. *p*-values less than 0.05 were considered statistically significant.

## 3. Results

### 3.1. Effect of Linoleic Acid and Fenofibrate on Body and Organ Weight

The body weights and the weights of the livers of the rats in the six study groups are shown in Table 2. No significant differences were observed between the body weight of the normal-diet-fed rats and the liver weights of the animals. However, the body weights of the rats in the hypercholesterolemic groups treated with linoleic acid (5 mg/kg) and fenofibrate (60 mg/kg) showed a significant difference (*p* < 0.05) in decreasing body weight compared with the control hypercholesterolemic rats. Other than that, neither supplementation with linoleic acid nor fenofibrate showed significant differences in the rat’s liver weights among all the study groups.

### 3.2. Effect of Linoleic Acid and Fenofibrate on Serum Lipid Profile and AI

**Figure 1:**
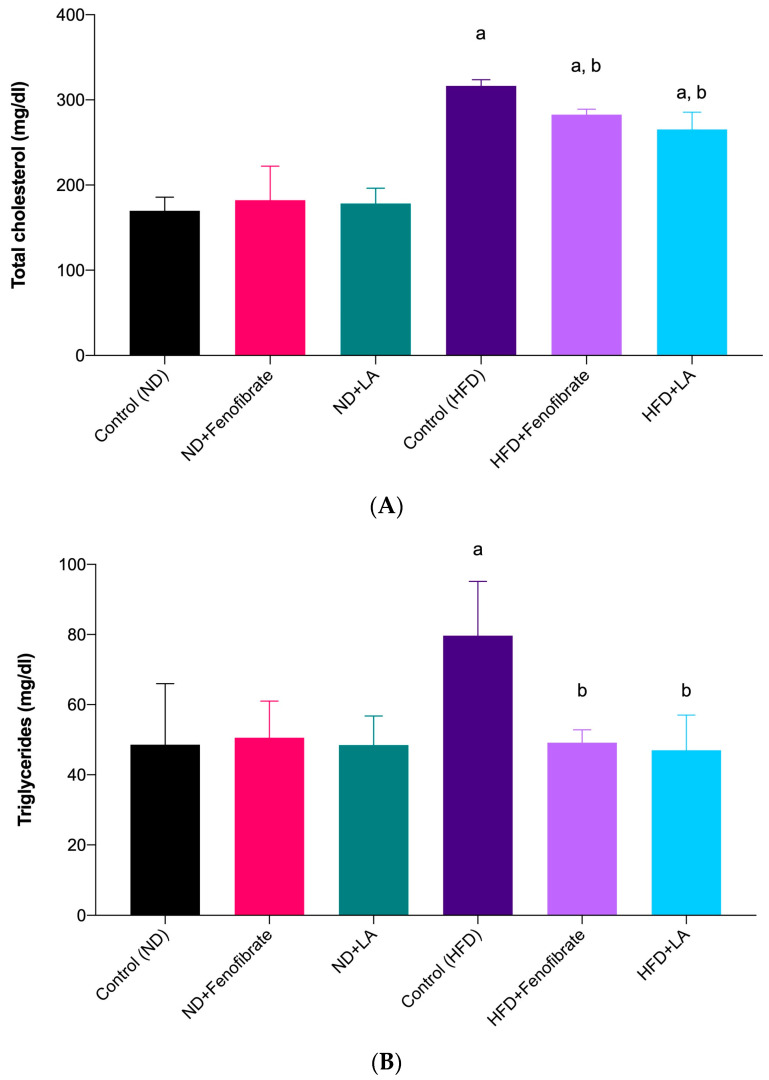

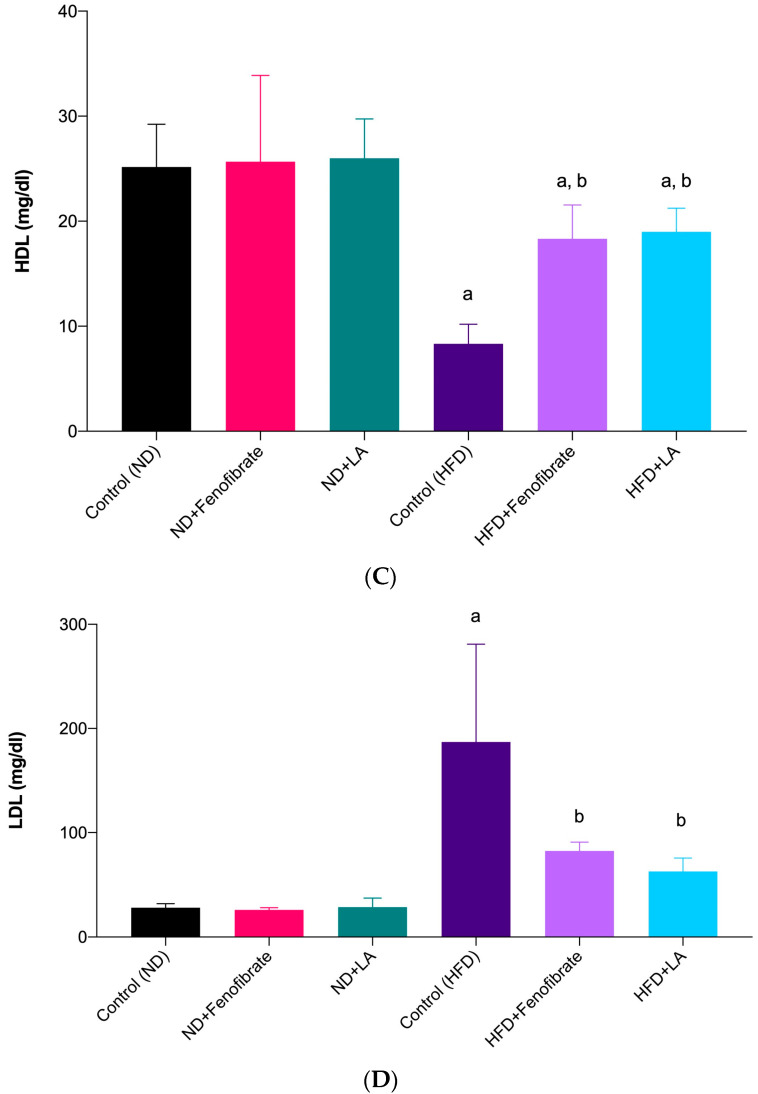
TC (**A**), TG (**B**), HDL (**C**), and LDL (**D**) levels in the serum of rats fed with a high-fat diet and a normal diet, treated with fenofibrate (60 mg/kg) and linoleic acid (5 mg/kg) for 28 days. Data are presented as mean ± SD (*n* = 6). ^a^ *p* < 0.05 vs. Control (ND); ^b^ *p* < 0.05 vs. Control (HFD). Normal diet: ND; high-fat diet: HFD; linoleic acid: LA; TC: total cholesterol: TC; triglycerides: TG; high-density lipoprotein: HDL; low-density lipoprotein: LDL.

The TC levels in the rats fed with a high-fat diet showed a significant decrease after 28 days of treatment with linoleic acid (HFD + LA: 265.50 ± 20.22 mg/dL vs. Control (HFD): 316.60 ± 7.30 mg/dL; *p* = 0.0069) and fenofibrate (HFD + Fenofibrate: 282.80 ± 6.25 mg/dL vs. Control (HFD): 316.60 ± 7.30 mg/dL; *p* = 0.0426) compared with the control hypercholesterolemic rats (Figure 1A). However, no statistical difference was observed in the groups fed with a normal diet.

In the present study, both hypercholesterolemic groups treated with linoleic acid (HFD + LA: 47.00 ± 10.05 mg/dL vs. Control (HFD): 79.67 ± 15.50 mg/dL; *p* = 0.0075) and fenofibrate (HFD + Fenofibrate: 49.17 ± 3.66 mg/dL vs. Control (HFD): 79.67 ± 15.50 mg/dL; *p* = 0.0104) for four weeks showed a statistical difference (*p* < 0.05) in TG levels compared with the control hypercholesterolemic group (Figure 1B). However, no significant difference was observed in the TG levels of the non-hypercholesterolemic groups treated with linoleic acid and fenofibrate when compared with the control non-hypercholesterolemic group. Moreover, no statistical difference was observed between the linoleic acid-treated and fenofibrate-treated groups in the hypercholesterolemic rats, suggesting the comparable effect of both treatments in reducing TG.

Figure 1C shows that the high-fat-diet-induced hypercholesterolemic rats treated with fenofibrate (HFD + Fenofibrate: 18.33 ± 3.22 mg/dL vs. Control (HFD): 8.33 ± 1.86 mg/dL; *p* = 0.0492) and linoleic acid (HFD + LA: 19.00 ± 2.24 mg/dL vs. Control (HFD): 8.33 ± 1.86 mg/dL; *p* = 0.0084) exhibited a significant increase in HDL levels compared with the control hypercholesterolemic rats (control). The result clearly shows that the rats fed with a high-fat diet without any treatment (control) showed a significant decrease in HDL levels. Apparently, this situation did not take place in the non-hypercholesterolemic groups (normal-diet-fed groups). No statistical difference was observed between the linoleic-acid-treated and fenofibrate-treated groups in the hypercholesterolemic rats, suggesting the comparable effect of both treatments in increasing HDL.

In the present study, both hypercholesterolemic groups treated with linoleic acid (HFD + LA: 62.83 ± 12.67 mg/dL vs. Control (HFD): 187.00 ± 93.95 mg/dL; *p* = 0.0005) and fenofibrate (HFD + Fenofibrate: 82.50 ± 8.27 mg/dL vs. Control (HFD): 187.00 ± 93.95 mg/dL; *p* = 0.0057) showed a statistically significant difference in LDL levels compared with the control hypercholesterolemic group (Figure 1D). The results show that LDL levels in the high-fat diet-induced hypercholesterolemic group reduced substantially when treated with 5 mg/kg of linoleic acid for four weeks. However, no statistical difference was observed in the normal diet-fed groups (non-hypercholesterolemic).

**Figure 2:**
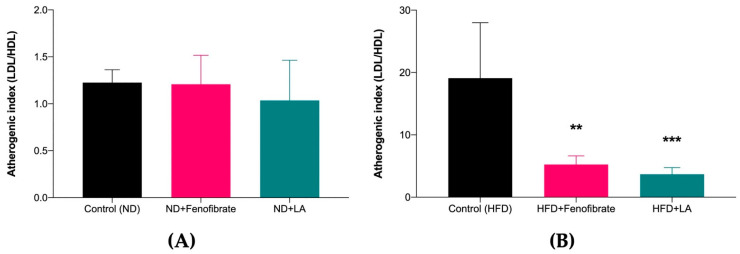
Atherogenic index of rats fed with normal diet (**A**) and high-fat diet (**B**) treated with linoleic acid (5 mg/kg) and fenofibrate (60 mg/kg) for 28 days. Data are presented as mean ± SD (*n* = 6). ** *p* < 0.01, *** *p* < 0.001 vs. Control (HFD). Normal diet: ND; high-fat diet: HFD; linoleic acid: LA; low-density lipoprotein: LDL; high-density lipoprotein: HDL.

Figure 2A shows that there was no significant difference among the study groups. However, Figure 2B shows that the AI in the hypercholesterolemic rats treated with linoleic acid (HFD + LA: 3.68 ± 1.06 vs. Control (HFD): 19.09 ± 8.90; *p* = 0.0009) and fenofibrate (HFD + Fenofibrate: 5.24 ± 1.38 vs. Control (HFD): 19.09 ± 8.90; *p* = 0.0035) showed a significant decrease compared with the control hypercholesterolemic rats.

### 3.3. Effect of Linoleic Acid and Fenofibrate on Liver Function Test

**Figure 3:**
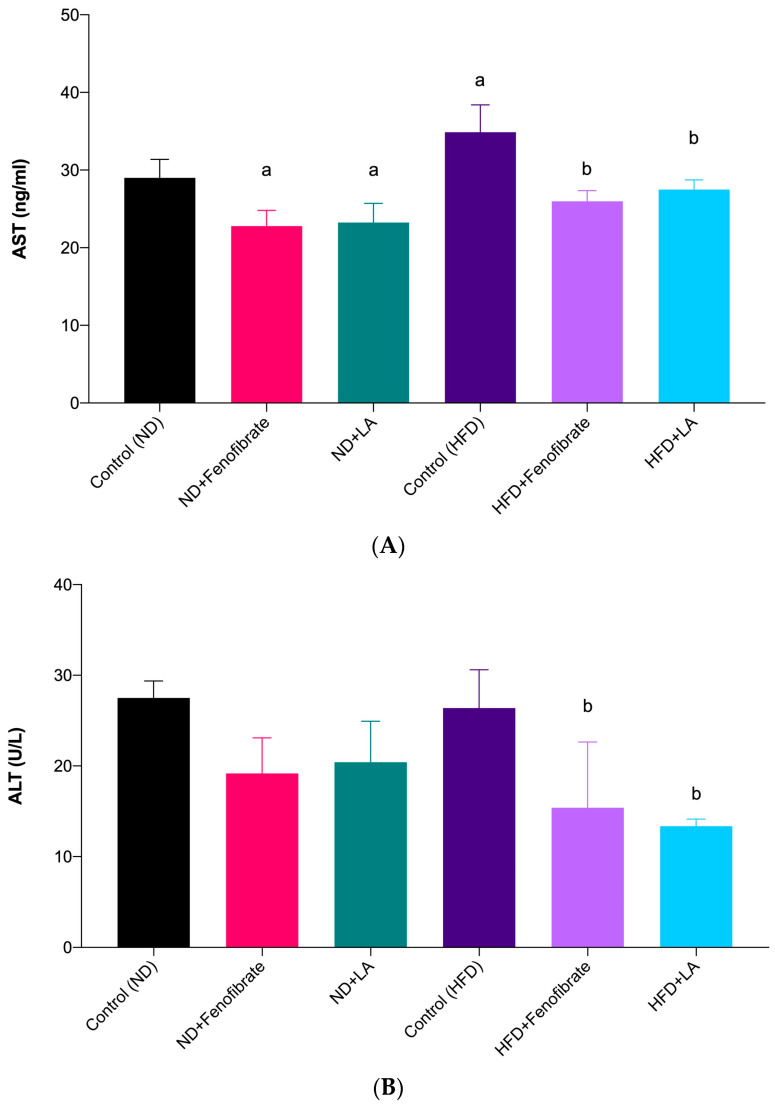
Serum AST levels of rats fed with a normal diet and a high-fat diet (**A**), and serum ALT levels of rats fed with a normal diet and a high-fat diet (**B**) treated with fenofibrate (60 mg/kg) and linoleic acid (5 mg/kg) for 28 days. Data are presented as mean ± SD (*n* = 6). ^a^ *p* < 0.05 vs. Control (ND); ^b^ *p* < 0.05 vs. Control (HFD). Normal diet: ND; high-fat diet: HFD; linoleic acid: LA; aspartate aminotransferase: AST; alanine aminotransferase: ALT.

In the present study, the AST levels in the non-hypercholesterolemic rats treated with linoleic acid (ND + LA: 23.24 ± 2.47 ng/mL vs. Control (ND): 29.01 ± 2.37 ng/mL; *p* = 0.0231) and fenofibrate (ND + Fenofibrate: 22.79 ± 2.01 ng/mL vs. Control (ND): 29.01 ± 2.37 ng/mL; *p* = 0.0132) showed a significant decrease compared with the control non-hypercholesterolemic rats (Figure 3A). In addition, the AST levels in the hypercholesterolemic rats treated with linoleic acid (HFD + LA: 27.50 ± 1.24 ng/mL vs. Control (HFD): 34.88 ± 3.52 ng/mL; *p* = 0.0107) and fenofibrate (HFD + Fenofibrate: 25.97 ± 1.39 ng/mL vs. Control (HFD): 34.88 ± 3.52 ng/mL; *p* = 0.0011) showed a significant decrease compared with the control hypercholesterolemic rats (Figure 3A).

Figure 3B shows that the ALT levels in the non-hypercholesterolemic rats treated with linoleic acid and fenofibrate exhibited no significant difference compared with the control non-hypercholesterolemic rats. However, the levels of ALT in the hypercholesterolemic rats treated with linoleic acid (HFD + LA: 13.36 ± 0.78 U/L vs. Control (HFD): 26.39 ± 4.21 U/L; *p* = 0.0096) and fenofibrate (HFD + Fenofibrate: 15.38 ± 7.26 U/L vs. Control (HFD): 26.39 ± 4.21 U/L; *p* = 0.0325) showed a significant decrease compared with the control hypercholesterolemic rats (Figure 3B).

### 3.4. Histopathological Changes in the Liver Tissue

**Figure 4:**
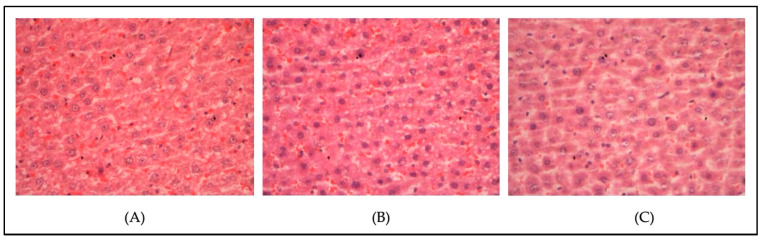
Histopathological changes in the liver of the non-hypercholesterolemic rats (magnification, ×400). (**A**) Control normal diet-fed rats; (**B**) normal diet-fed rats treated with fenofibrate (60 mg/kg); (**C**) normal diet-fed rats treated with linoleic acid (5 mg/kg).

**Figure 5:**
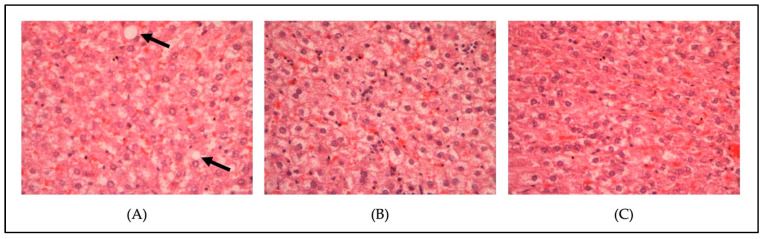
Histopathological changes in the liver of the hypercholesterolemic rats (magnification, ×400). (**A**) Control high-fat diet-fed rats; (**B**) high-fat diet-fed rats treated with fenofibrate (60 mg/kg); (**C**) high fat diet-fed rats treated with linoleic acid (5 mg/kg). The black arrow shows cholesterol deposits within the cytoplasm.

**Figure 6:**
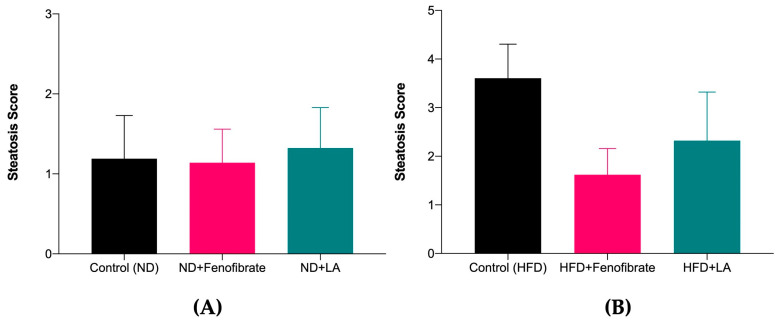
Steatosis scoring of liver tissue of rats fed with a normal diet (**A**) and a high-fat diet (**B**), treated with fenofibrate (60 mg/kg) and linoleic acid (5 mg/kg) for 28 days. Data are presented as mean ± SD (*n* = 6). Normal diet: ND; high-fat diet: HFD; linoleic acid: LA.

Figure 4 and Figure 5 show the histopathological findings of the liver tissue of a normal diet-fed rat (non-hypercholesterolemic) (Figure 4) following treatment with linoleic acid and fenofibrate, and a high-fat-diet-induced hypercholesterolemic rat following treatment with linoleic acid (5 mg/kg) and fenofibrate (60 mg/kg) (Figure 5). The tissue section of the normal group (Control, Figure 4A) showed the normal liver architecture indicated by normal hepatic cells with the characteristic morphology of a well-preserved cytoplasm, a prominent nucleus, and sinusoidal spaces. However, the tissue section of the liver of the control hypercholesterolemic rats demonstrated severe architectural damage specified by hemorrhage, steatosis, and cholesterol deposition in the cytoplasm (Figure 5A). Treatment with linoleic acid (Figure 4C and Figure 5C) and fenofibrate (Figure 4B and Figure 5B) reduced architectural damage and cholesterol deposition in the liver. The histopathology slides were subjected to a semi-quantitative analysis, where the steatosis was graded on a scale of one to four, with respect to the presence of the cholesterol droplets. The steatosis was compared between the treated and control groups. Figure 6 shows no significant differences (*p* > 0.05) in the liver steatosis score when comparing the hypercholesterolemic groups treated with linoleic acid (5 mg/kg) with the control hypercholesterolemic group.

## 4. Discussion

The experiment was designed to determine the effect of linoleic acid on reducing hypercholesterolemia effects in comparison with fenofibrate in rats fed with a high-fat diet for 28 days. The current study was carried out to compare the effectiveness of linoleic acid treatment in reducing hypercholesterolemia by increasing HDL and reducing LDL levels in plasma cholesterol levels. The current findings showed that the administration of 5 mg/kg of linoleic acid improved TC, TG, HDL, and LDL levels in a high-fat-diet-induced hypercholesterolemic rat model. The present study also demonstrated that treatment with linoleic acid reduced the AI, serum AST, and ALT levels in the hypercholesterolemic rats compared with the control hypercholesterolemic rats. Furthermore, this study demonstrated that oral treatment with linoleic acid showed mild structural changes by lowering cholesterol droplets deposited into the cytoplasm in the liver tissue of the hypercholesterolemic rats.

In the present study, all the animals showed a trend of increase in their body weight at the end compared with that at the beginning of the experiment. Previously, it has been observed that conjugated linoleic acid reduces body fats and increases lean mass [46]. However, there are other reported cases which found no effect or an increase in body weight [47]. The precise effect of linoleic acid is a much-debated topic, as it has been associated with an increase in energy expenditure. However, in the present study, body weight gain was slightly affected. Treatment with linoleic acid in the hypercholesterolemic rats led to a significant reduction in their body weight compared with the control hypercholesterolemic rats. This finding was in line with previous findings reported by Banu et al. [48] and Kanaya and Chen [49]. Evidence shows that weight loss has a significant impact on health improvement, suggesting that a modest weight loss of 5% to 10% of the initial body weight can occur in parallel with effective treatment [50]. Previously, some animal studies have reported that supplementation with PUFA may lead to weight loss and reduced fat mass in mice [51], whereas others have shown PUFA does not have significant weight loss effects [52]. The factors that could influence weight loss through a diet supplement with PUFA have been explored in several studies. Conflicting reports on the effect of linoleic acid in several studies may be related to the energy level of the diet itself. The consumption of unsaturated fatty acid downregulates lipogenesis-related genes and upregulates fatty acid oxidation-related genes, thereby inducing a reduction in serum TG, fatty acid levels, fatty acid cellular uptake, and the extent of fat deposition [53].

In relation to the liver weights of the rats in the present study, no significant difference was observed between all the hypercholesterolemic groups compared with the control; the results were confirmed when this parameter was taken in relation to the body weight. These results indicate that there is no induction of fatty generation in the liver. Thus, the hypolipidemic effect of linoleic acid treatment may not be due to the distribution of lipids from the plasma to the liver but rather, to the lower intestinal absorption of lipids or higher lipid catabolism [54].

The mechanism that leads to the induction of atherogenesis was shown to be a result of an accumulation of lipids within the arterial wall. A high plasma level of LDL cholesterol is the main risk factor for atherosclerosis and leads to cardiovascular diseases [27,30]. The present study has confirmed the effectiveness of the high-fat diet, in combination with 1% cholesterol and 0.1% cholic acid, consumed by the rats for 42 days, effectively increasing the TC, TG, and LDL levels in the rats’ blood serum. Cholesterol in the cholic acid diet provides a high level of dietary cholesterol, and the intestinal absorption of this cholesterol is several times higher in the presence of cholic acid [55]. Previous research has established that rats fed with a diet supplemented with high cholesterol alone do not develop hypercholesterolemia [56]. However, combining it with cholic acid showed an increased risk of developing atherosclerosis. The additional combination with a normal diet has been demonstrated to increase serum cholesterol and lipoprotein levels [57]. Lipids are generated when LDL undergoes an oxidative breakdown of phospholipids. This highly reactive oxidized LDL plays a pathogenic role in the development of atherosclerosis. Several studies have reported findings similar to those in the current study and suggest that prolonged high serum cholesterol levels increase the risk of developing atherosclerosis [27,55,58,59].

In the present study, the administration of linoleic acid in the high-fat-diet-induced hypercholesterolemic rats significantly reduced TC, TG, and LDL and increased HDL levels compared with those in the control hypercholesterolemic rats. The effects of linoleic acid were comparable with the effects of fenofibrate in the hypercholesterolemic rats. The present findings were in line with previous studies on the effects of linoleic acid [18,19,60,61]. The effects of linoleic acid in reducing lipid levels were comparable with the effects of fenofibrate. However, treatment with linoleic acid and fenofibrate in the non-hypercholesterolemic rats did not show any significant difference compared with the control non-hypercholesterolemic rats. Evidence shows that the activation of the PPARα pathway in the liver is responsible for the lipid-lowering action [62]. PPARα is a ligand-activated transcription factor that controls a comprehensive set of genes involved in most aspects of lipid catabolism. An extensive study has demonstrated that the induction of *SR-B1* genes facilitates a bidirectional flux of free cholesterol between cells and lipoproteins in response to a PPAR agonist and also contributes to reverse cholesterol transport [63].

The current study has demonstrated that treatment with linoleic acid in hypercholesterolemic rats significantly reduced the serum AST and ALT levels compared with those in the control hypercholesterolemic rats. In addition, treatment with linoleic acid in the non-hypercholesterolemic rats significantly reduced the serum AST levels compared with those of the control non-hypercholesterolemic rats. These findings are in line with the results of previous studies [64,65,66].

Hepatic liver steatosis is defined as the presence of intrahepatic fat in amounts of at least 5% of the liver weight. Simple accumulation of TG in the liver can be hepatoprotective; however, prolonged hepatic lipid storage may lead to liver metabolic dysfunction [45]. Hepatic steatosis is a reversible condition that can be corrected by lifestyle modifications, such as physical activity and dietary intervention. Fatty disorders of the liver can be classified into alcoholic and non-alcoholic. Non-alcoholic is defined as the presence of hepatic steatosis without evidence of hepatocellular injury in the form of the ballooning of the hepatocyte, whereas alcoholic fatty liver involves inflammation and hepatocellular injury [67]. The qualitative analysis showed that cholesterol droplets could be found within and in between the cells. Based on the physical observation under ×400 magnification using a light microscope, there were more cholesterol droplets found in the control hypercholesterolemic groups compared with the treated groups. The current study also demonstrated that the hypercholesterolemic rats treated with linoleic acid and fenofibrate showed mild cholesterol droplets in their liver tissues. However, no significant difference was observed between the steatosis scoring among all the study groups. The effect in reducing cholesterol droplets in the liver tissue may be due to the reducing effect of linoleic acid and fenofibrate on serum LDL levels. This effect was supported by the increased HDL levels in both hypercholesterolemic-treated groups.

Since the present study demonstrated the lipid-lowering effects of linoleic acid treatment in hypercholesterolemic rats, there are a few limitations in the present study. Firstly, in regards to the cholesterol levels in serum hypercholesterolemic rats treated with linoleic acid and fenofibrate, the atherosclerosis parameters were not measured in the current study. The atherogenic parameters include histology of the blood vessels, particularly the thoracic aorta (localization of the foam cells and intima-media thickness), the vascular tissue inflammation marker (tumor necrosis factor-alpha: TNF-α; interleukins-1: IL-1, and IL-6), anthropometric measurement (body mass index and Lee’s index), serum insulin levels, and homeostatic model assessment for insulin resistance (HOMA-IR). Secondly, the present study only focused on the effects of linoleic acid and fenofibrate on the serum cholesterol levels, rather than on the levels of *SR-BI* gene expression, SR-BI protein expression, and HDL-uptake assay for the tissue of the hypercholesterolemic rats. These parameters were not evaluated, due to budget constraints. Therefore, future studies on the effects of linoleic acid and fenofibrate on the above parameters are suggested to identify the effectiveness of linoleic acid in reducing cholesterol levels and preventing atherosclerosis development in obese or hypercholesterolemia models.

## 5. Conclusions

The present study showed that the administration of linoleic acid for four weeks improved the serum lipid profile, particularly TC, TG, HDL, and LDL levels, reduced AI, AST, and ALT, and engendered mild structural changes in the liver tissues of a high-fat-diet-induced hypercholesterolemic rat model. These effects were comparable to those of fenofibrate, indicating that linoleic acid has the potential to be an adjunct in the treatment of hypercholesterolemia to prevent or reduce the severity of atherosclerosis development.

## Figures and Tables

**Table 1:** Grouping of the experimental animals based on diet and treatment.

Group	Sub-Group	Details
Non-hypercholesterolemic	1	Control normal diet-fed rats (Control (ND))
	2	Normal diet-fed rats treated with 60 mg/kg daily of fenofibrate (ND + Fenofibrate)
	3	Normal diet-fed rats treated with 5 mg/kg daily of linoleic acid (ND + LA)
Hypercholesterolemic	1	Control high-fat diet-fed rats (Control (HFD))
	2	High-fat diet-fed rats treated with 60 mg/kg daily of fenofibrate (HFD + Fenofibrate)
	3	High-fat diet-fed rats treated with 5 mg/kg daily of linoleic acid (HFD + LA)

**Table 2:** Body weight and liver weight of rats fed with a normal diet and a high-fat diet.

	Control	Fenofibrate (60 mg/kg)	Linoleic Acid (5 mg/kg)
Normal Diet			
Initial body weight (Week 0), (g)	269.40 ± 30.32	281.80 ± 16.09	273.30 ± 42.63
Final body weight (Week 6), (g)	291.40 ± 32.34	298.40 ± 20.53	293.90 ± 41.80
High-fat diet			
Initial body weight (Week 0), (g)	278.10 ± 25.44	268.40 ± 11.99	267.90 ± 26.38
Final body weight (Week 6), (g)	339.90 ± 15.37	282.80 ± 6.25 *	274.10 ± 23.92 **
Normal diet			
Liver weight (g)	2.68 ± 0.76	3.55 ± 1.67	2.76 ± 0.38
High-fat diet			
Liver weight (g)	3.24 ± 0.36	3.02 ± 0.58	2.99 ± 0.40

Data were expressed as mean ± SD (*n* = 6). * p < 0.05, ** *p* < 0.01 vs. Control group.

## Data Availability

Data is available upon request.
